# Long-Term Enzyme Replacement Therapy and Renal Outcomes in Fabry Disease: A Systematic Review and Meta-Analysis

**DOI:** 10.3390/biomedicines13122989

**Published:** 2025-12-05

**Authors:** Chih-Yang Chen, Chun-Ting Li, Cheng-Jui Lin, Hong-Mou Shih, Shu-Jung Liu, Fang-Ju Sun, Chih-Jen Wu

**Affiliations:** 1 Division of Nephrology, Department of Internal Medicine, MacKay Memorial Hospital, Taipei City 104217, Taiwan; lorgenchen11@gmail.com (C.-Y.C.); yesgshm@gmail.com (H.-M.S.); 2 Division of Nephrology, Department of Internal Medicine, Landseed International Hospital, Taoyuan City 324609, Taiwan; yumetihiro@gmail.com; 3 Department of Medicine, Mackay Medical College, New Taipei City 251020, Taiwan; 4 Department of Medical Library, MacKay Memorial Hospital, Tamsui Branch, New Taipei City 251020, Taiwan; 5 Department of Medical Research, MacKay Memorial Hospital, Taipei City 104217, Taiwan

**Keywords:** Fabry disease, enzyme replacement therapy (ERT), systematic review, meta-analysis, glomerular filtration rate (GFR), proteinuria, renal outcomes, clinical events, nephropathy

## Abstract

**Background**: Fabry disease is a rare X-linked lysosomal storage disorder associated with progressive renal, cardiac, and neurological complications. Enzyme replacement therapy (ERT) has been the standard treatment for more than two decades, but its long-term impact on renal outcomes remains debated. **Methods**: We conducted a systematic review and meta-analysis of studies reporting renal outcomes in Fabry patients under long-term follow-up, including both ERT-treated and untreated cohorts. Electronic databases were searched up to October 2023. Data were extracted on estimated glomerular filtration rate (eGFR) slope, proteinuria, and clinical events. Random-effects models were used to calculate pooled effect sizes, and subgroup analyses were performed by treatment status and baseline risk factors. **Results**: Sixteen studies involving 2191 patients were included. Pooled analyses demonstrated a significant decline in eGFR over time across Fabry cohorts. Crucially, baseline proteinuria was identified as a significant prognostic factor; male patients with baseline UPCR > 0.5 g/g experienced a significantly faster decline in eGFR compared to those with UPCR < 0.5 g/g (*p* = 0.011). While direct comparisons between ERT and non-ERT groups did not consistently reach statistical significance, trends suggested a slower decline in ERT-treated patients, particularly in those with preserved renal function and lower proteinuria. Patients with baseline eGFR < 60 mL/min/1.73 m^2^ had a significantly higher risk of clinical events compared with those with preserved renal function. **Conclusions**: Fabry patients experience progressive renal decline despite available therapies. Although direct comparisons between ERT and non-ERT groups did not consistently reach statistical significance, our quantitative analysis highlighted baseline proteinuria as a major determinant of renal trajectory. Patients with baseline UPCR > 0.5 g/g exhibited a significantly faster decline in eGFR, emphasizing the importance of early diagnosis and intervention before significant glomerular damage occurs. The limitations of the analysis include the small number of studies, heterogeneity in renal function definitions, exclusion of advanced kidney disease, and methodological constraints related to effect size reporting and risk-of-bias assessment.

## 1. Introduction

Fabry disease is a rare, inherited disorder caused by a genetic defect that affects the enzyme alpha-galactosidase A (α-Gal A). It affects approximately 1 in 4000 to 170,000, with newborn screening studies suggesting that the disease is significantly underdiagnosed and more prevalent than historically estimated [1,2]. As a lysosomal storage disorder, it leads to various symptoms and complications, including pain in the hands and feet, fatigue, kidney failure, stroke, and heart problems [3,4]. Fabry disease was first described in the late 19th century in a family with a history of early death due to a mysterious illness. However, the genetic basis of Fabry disease—that pathogenic variants in the gene encoding α-Gal A cause the disorder—was not discovered until the 1960s [5]. Since then, researchers have identified more than 1000 variants in the GLA gene, including those associated with classic and later-onset phenotypes [1]. Based on our previous statistical data [6], the prevalence of Fabry disease in chronic kidney disease (CKD) patients is higher than expected, but the disease is often overlooked for various reasons.

The symptoms of Fabry disease can be moderate to severe and vary in their clinical appearance. The most common symptoms develop in childhood or adolescence, including neuropathic pain, angiokeratoma, paresthesia, and corneal opacities [3,4,7]. Several organ systems, most notably the cardiovascular, kidney, and central nervous systems, are affected in Fabry disease. The effects are mediated by the gradual accumulation of globotriaosylceramide (Gb3) in cells [3,4,7,8]. One of the critical characteristics of Fabry disease is progressive nephropathy, which has an insidious onset and an overall rate of chronic kidney disease (CKD) progression [9,10,11,12,13]. Renal function can gradually deteriorate from a young age, leading to kidney failure and the need for dialysis or a kidney transplant in later life. Furthermore, the poorer the renal function, the greater the simultaneous organ dysfunction. In some studies, deterioration of renal function is regarded as disease progression [11,12,13].

Enzyme replacement therapy (ERT) involves administering a recombinant form of α-Gal A to patients; however, this can only slow disease progression not cure the disease [14,15,16]. The first clinical trials involving ERT for Fabry disease were conducted in the late 1990s, and the regimen has been commercially available since 2001 [14,15,16]. There are two forms available. Agalsidase alpha (Replagal^®^) is administered via intravenous infusion at 0.2 mg/kg every other week (EOW), whereas agalsidase beta (Fabrazyme^®^) is administered via intravenous infusion at 1.0 mg/kg/EOW. Compared to receiving no treatment, the administration of agalsidase is believed to delay estimated glomerular filtration rate (eGFR) decline [15]. Although pivotal clinical trials included patients with impaired renal function [17,18], the long-term effectiveness of ERT in stabilizing renal function—particularly in patients with advanced nephropathy—remains a critical area of investigation regarding the potential ‘point of no return’. The treatment of Fabry disease is constantly evolving, including the use of chaperone therapy and novel pegylated recombinant alpha-GAL A, with many of these drugs often compared to enzyme replacement therapy (ERT) as a control group. Therefore, the effectiveness of ERT in patients with impaired kidney function requires specific research and considered discussion. Nevertheless, there remains much to learn about the long-term effects of ERT on the risk of clinical events and renal outcomes in individuals with varying baseline renal function. To our knowledge, no previous meta-analysis has specifically addressed the long-term renal outcomes of ERT in Fabry disease, nor comprehensively evaluated how baseline renal function and proteinuria modify prognosis. Therefore, we conducted a systematic review and meta-analysis to evaluate the impact of ERT on renal decline and clinical outcomes in Fabry patients, with particular attention to baseline risk stratification.

## 2. Materials and Methods

### 2.1. Eligibility Criteria

We included studies that enrolled patients with genetically or clinically confirmed Fabry disease and that reported renal outcomes over long-term follow-up. Eligible cohorts included patients receiving enzyme replacement therapy (ERT) or untreated patients (natural history cohorts). When available in the same study, both treated and untreated groups were retained to allow for comparative analyses. Studies had to provide sufficient data on at least one of the following outcomes: estimated glomerular filtration rate (eGFR) slope, urinary protein/creatinine ratio (UPCR) slope, or renal-related clinical events (composite of kidney failure, cardiovascular complications, or death). Studies with overlapping populations were carefully screened; in such cases, the dataset with the longest follow-up and most comprehensive reporting was included. This systematic review and meta-analysis was conducted in accordance with the PRISMA 2020 reporting guidelines. The review protocol was retrospectively registered in the INPLASY database (registration number: INPLASY2025100029). All checklist items were addressed, and the completed PRISMA 2020 checklist is provided in Supplementary Table S1.

### 2.2. Search Strategy

This systematic review and meta-analysis followed the Preferred Reporting Items for Systematic Reviews and Meta-Analyses (PRISMA) guidelines. No protocol was published in advance. PubMed, Cochrane CDSR, Cochrane CENTRAL, EMBASE, and Airiti Library were searched from inception to 13 October 2023. Searches were restricted to human studies published in English. The search strategy used a combination of keywords related to Fabry disease and ERT to maximize the search results. The search strategy for PubMed was: (Fabry disease) AND (enzyme replacement therapy OR Agalsidase alpha OR Replagal OR Agalsidase beta OR Fabrazyme).

Reference lists of included studies were hand-searched to identify other potentially relevant studies.

The inclusion criteria were RCTs, retrospective studies, or prospective studies focused on adults with Fabry disease receiving ERT and reporting at least one quantitative outcome of interest. Duplicate publications, studies with no quantitative outcomes of interest, letters, commentaries, editorials, proceedings, case reports, personal communications, or studies that analyzed the same patient sample as preexisting research were excluded.

### 2.3. Study Selection

Two reviewers (CY Chen and CT Li) independently screened all titles and abstracts retrieved from the database search. Full texts of potentially eligible articles were then assessed in detail against the prespecified inclusion and exclusion criteria. Any disagreements were resolved by discussion and consensus, with arbitration by a third reviewer (CJ Wu) when necessary. Reasons for exclusion at the full-text stage were documented. The overall selection process is summarized in the PRISMA flow diagram. The PRISMA 2020 checklist is available in Supplementary Table S1.

### 2.4. Main Outcome Measures

The primary outcome was the yearly change in eGFR (i.e., eGFR slope). The secondary outcome was the occurrence of clinical events, including death, cardiac events (i.e., symptomatic arrhythmia and myocardial infarction), renal events (i.e., progression of CKD, new onset of dialysis or kidney transplantation), and neurologic events (i.e., stroke or transient ischemic attack [TIA]).

Study eligibility was determined by two independent reviewers based on the above strategy and criteria. When there was uncertainty regarding eligibility, a third reviewer was consulted.

### 2.5. Data Extraction

Data from each eligible study were independently extracted by two reviewers (CY Chen, CT Li) using a standardized data collection form. Extracted variables included study characteristics (first author, publication year, country, study design, sample size, follow-up duration), patient demographics (age, sex distribution), clinical parameters (baseline eGFR, proteinuria, treatment status), and outcomes of interest (annual eGFR slope, UPCR changes, incidence of renal or clinical events). When studies reported both ERT-treated and untreated cohorts, data from each group were extracted separately. For overlapping publications derived from the same cohort, the dataset with the longest follow-up and most comprehensive reporting was selected. Any discrepancies in data extraction were resolved by consensus.

### 2.6. Quality Assessment

As previously described, the Quality in Prognosis Studies (QUIPS) tool assessed bias in any of the included studies. This tool evaluates bias in any of six domains within a study, including study participants, study attrition, prognostic factor measurement, outcome measurement, study confounding, and statistical analysis and reporting. Two independent reviewers (Li CT and Lin CJ) performed a quality assessment, and disagreement was resolved through discussion. Assessed outcomes for the included studies are shown in Supplementary Figure S1.

### 2.7. Statistical Analysis

All analyses were performed using Comprehensive Meta-Analysis software, version 3.0 (Biostat, Englewood, NJ, USA). Standardized mean differences (SMDs) with 95% confidence intervals (CIs) between comparison groups were calculated and compared for continuous outcomes. For outcomes reported as longitudinal slopes (e.g., annual eGFR change), each cohort contributed a single within-group estimate. These slopes were pooled as single-arm continuous outcomes and therefore do not represent direct head-to-head comparisons between treated and untreated patients. To examine whether treatment exposure modified the between-study differences in slopes, a meta-regression model was performed with ERT status included as a moderator. Summary effects were derived, and a 2-sided *p*-value < 0.05 between the comparison groups was considered statistically significant. A χ^2^-based test of homogeneity was performed, and the inconsistency index (I2) and Q statistics were determined. If I2 was >50% or >75%, the trials were considered heterogeneous or highly heterogeneous, respectively. If I2 was <25%, the studies were considered homogeneous. There are two statistical models for meta-analysis: the fixed-effects and random-effects models. The fixed-effects model depends on the hypothesis that all studies in the meta-analysis share a true effect size. In contrast, in the random-effects model, the true effect size may differ from study to study. When the power of the heterogeneity test was insufficient, a random-effects model was applied. The fixed-effects model was used if significant heterogeneity was not found [18]. We did not perform traditional funnel plots or formal tests for small-study effects on analyses comprising fewer than five studies (k < 5) due to insufficient statistical power to detect asymmetry. However, to evaluate the robustness of our overall findings, a leave-one-out sensitivity analysis was performed to examine the stability of the pooled results by excluding one study at a time. Furthermore, based on recent methodological guidance, we assessed publication bias, or small-study effects, for all pooled syntheses comprising at least five studies (k ≥ 5). We constructed the Doi plots and calculated the corresponding Luis Furuya–Kanamori (LFK) index [19]. These calculations were performed using custom scripts executed in the R statistical environment (version 4.5.2), developed based on established methodological definitions and algorithms.

## 3. Results

### 3.1. Search Result

**Figure 1:**
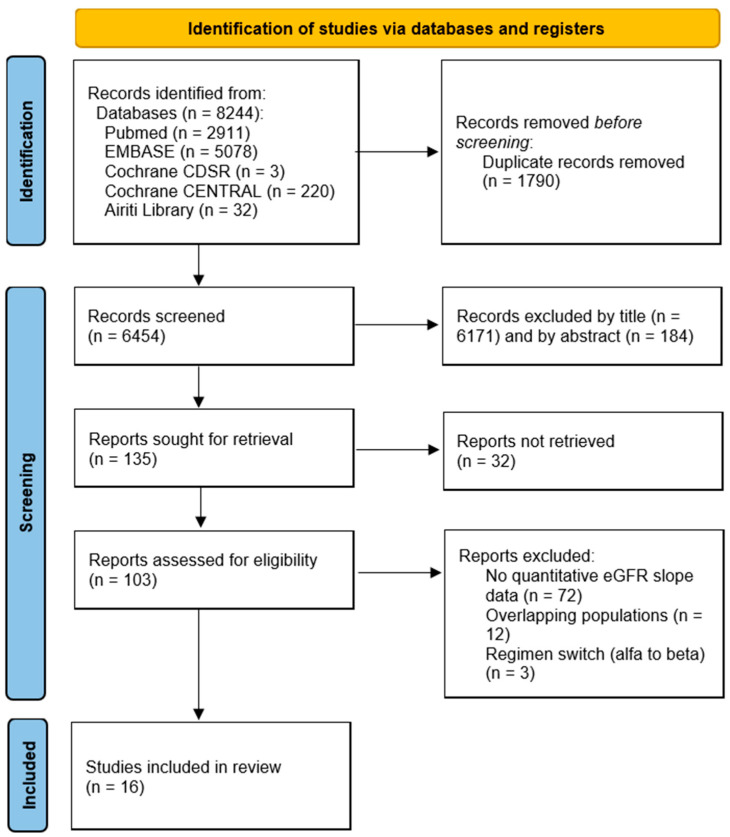
PRISMA 2020 flow diagram of study selection.

A flowchart of the search and study selection [20] process is shown in Figure 1. The electronic search identified 8244 unique records. After title and abstract screening, 8141 were excluded, and 135 were retrieved for full-text review. Of these 135 reports, 32 could not be retrieved because they were conference abstracts with no available full text. Ultimately, 16 studies met the eligibility criteria.

### 3.2. Study Characteristics

Among the included studies, most evaluated Fabry patients treated with enzyme replacement therapy (ERT), while several also reported untreated (natural history) cohorts. This allowed us to conduct both pooled analyses of overall renal progression and subgroup comparisons between ERT and non-ERT patients. Table 1 summarizes the study design, patient demographic characteristics, baseline eGFR, type of ERT, and follow-up duration. A total of 2191 patients were included. The patient number ranged from 4 to 560 across the studies. The proportion of males ranged from 50% to 100%. The mean follow-up duration in the included studies was 53 weeks to 8.1 years.

### 3.3. Risk of Bias of Included Studies

Quality assessment results for the included studies are shown in Supplementary Figure S1. All included studies had a low risk of bias in study confounding. Most studies had a low risk of bias in study participation, prognostic factor measurement, outcome measure, and statistical analysis and reporting. In general, the quality of the included studies was good.

### 3.4. Annual eGFR Decline Across Cohorts and Meta-Regression by Treatment Status

**Figure 2:**
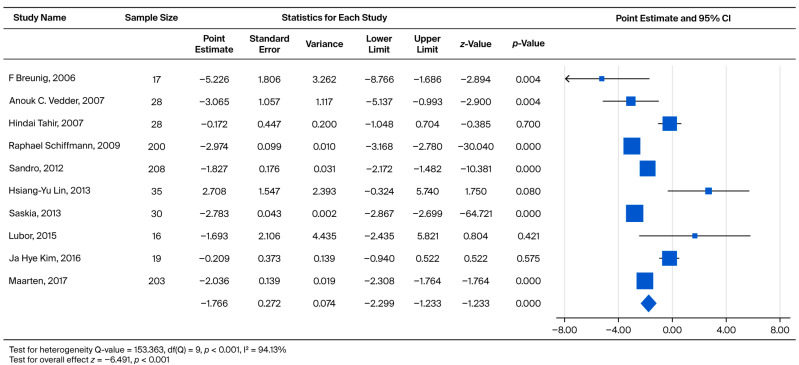
Forest plot comparing the annual eGFR slope between the ERT-treated and untreated (non-ERT) groups. A negative effect size indicates a faster eGFR decline in the untreated (non-ERT) group compared to the ERT-treated group [18,21,22,23,26,27,29,30,31,35].

Across all included cohorts, the pooled annual eGFR slope was −1.766 mL/min/1.73 m^2^/year (*p* < 0.001), indicating progressive renal decline among Fabry patients regardless of treatment exposure. As shown in Figure 2, the forest plot summarizes the within-study slopes reported by each cohort; these estimates represent longitudinal eGFR change within individual studies rather than direct comparisons between treated and untreated groups.

To further determine whether treatment status contributed to differences in slopes across studies, a meta-regression model was conducted. In Table 2, the intercept (−2.974, *p* = 0.010) reflects the pooled mean yearly decline across all cohorts. The regression coefficient for “ERT vs. no ERT” (+1.555, *p* = 0.210) indicates that treatment status alone did not significantly account for the observed between-study heterogeneity in eGFR slopes.

**Figure 3:**
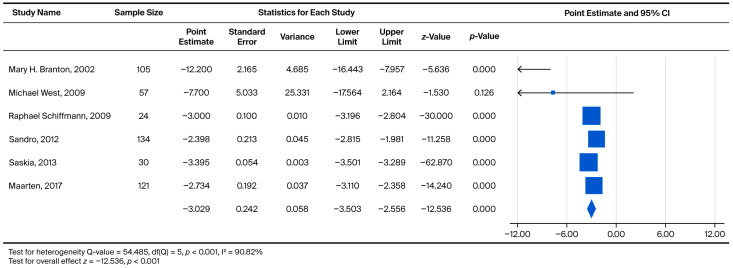
eGFR decline in ERT-treated and non-ERT-treated men [8,18,24,26,27,31].

Among male Fabry patients, the pooled annual eGFR slope was −3.029 mL/min/1.73 m^2^/year (*p* < 0.001), indicating a steeper rate of renal decline compared with the overall population. As illustrated in Figure 3, each point estimate represents the within-study yearly slope reported for male cohorts, reflecting longitudinal eGFR changes within individual studies rather than direct head-to-head comparisons between treated and untreated male patients.

To examine whether treatment status accounted for variability in eGFR slopes among male cohorts, a meta-regression model was applied. As shown in Table 3, the intercept (−3.532, *p* < 0.001) represents the pooled mean yearly decline in male patients. The regression coefficient for “ERT vs. no ERT” was +0.508 (*p* = 0.317), suggesting that treatment status alone did not significantly explain between-study differences in eGFR slope among male cohorts.

### 3.5. eGFR Decline Compared with eGFR > 60 and eGFR < 60

**Figure 4:**
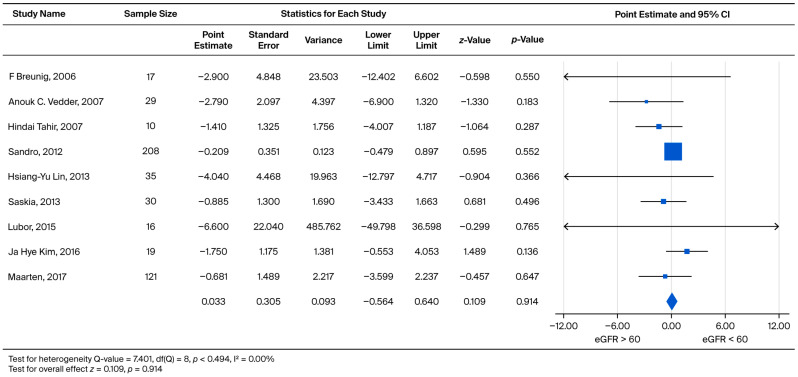
Subgroup analysis comparing the annual eGFR slope between patients with baseline eGFR ≥ 60 mL/min/1.73 m^2^ and eGFR < 60 mL/min/1.73 m^2^. A negative effect size indicates a faster eGFR decline in the eGFR < 60 mL/min/1.73 m^2^ group [21,22,23,26,27,29,30,31,35].

In order to compare the rate of subsequent eGFR decline, male and female patients were grouped together into two categories: eGFR > 60 (CKD1 + CKD2) and eGFR < 60 (CKD3 + CKD4 + CKD5). We subtracted the eGFR slope of eGFR > 60 from the eGFR < 60 to obtain an effect size. The effect sizes for standardized measures ranged from −6.600 to 1.750, with an overall random weighted mean effect size of 0.033, which was not significantly different from 0 (*p* = 0.914) (Figure 4).

**Figure 5:**
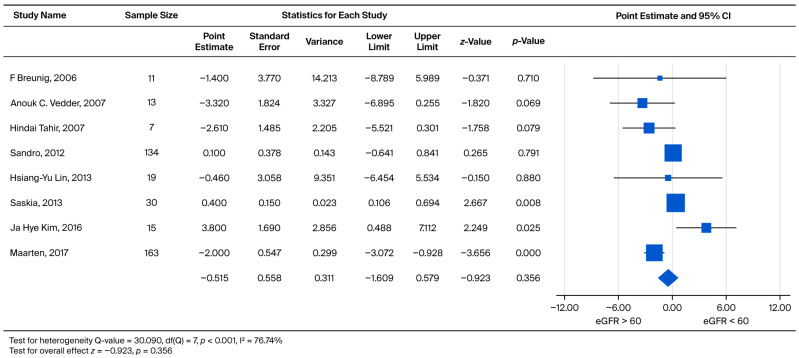
Subgroup analysis of annual eGFR slope in male patients: comparison between those with baseline eGFR ≥ 60 and eGFR < 60 mL/min/1.73 m^2^. A negative effect size indicates a faster eGFR decline in the eGFR < 60 mL/min/1.73 m^2^ group [21,22,23,26,27,30,31,35].

In order to further investigate the rate of subsequent eGFR decline, male patients were separated into two categories: eGFR > 60 (CKD1 + CKD2) and eGFR < 60 (CKD3 + CKD4 + CKD5). We subtracted the eGFR slope of eGFR > 60 from the eGFR < 60 to obtain an effect size. The effect sizes for standardized measures ranged from −3.320 to 3.800, with an overall random weighted mean effect size of −0.515, which was not significantly different from 0 (*p* = 0.356) (Figure 5).

### 3.6. UPCR Increase Compared with eGFR > 60 and eGFR < 60

**Figure 6:**
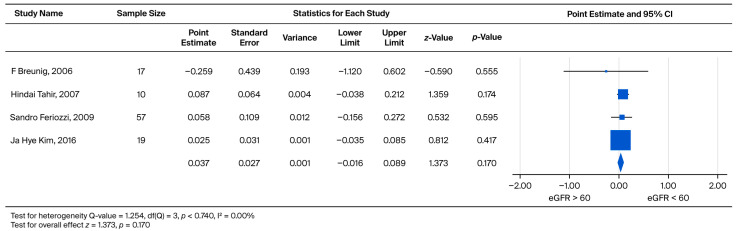
Subgroup analysis of annual UPCR change in the overall cohort: comparison between patients with baseline eGFR ≥ 60 and eGFR < 60 mL/min/1.73 m^2^. A positive effect size indicates a greater increase in UPCR in the eGFR < 60 mL/min/1.73 m^2^ group [21,23,26,30].

The subsequent rate of UPCR increase was also evaluated by comparing the two eGFR categories (eGFR > 60 vs. eGFR < 60) across the combined cohort. The standardized effect sizes were found to range from −0.259 to 0.087. This pooled analysis yielded a weighted mean effect size of 0.037, which did not reach statistical significance (*p* = 0.170) (Figure 6).

**Figure 7:**
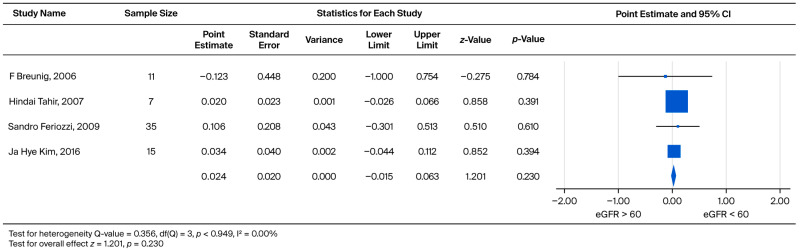
Subgroup analysis of annual UPCR change in male patients: comparison between those with baseline eGFR ≥ 60 and eGFR < 60 mL/min/1.73 m^2^. A positive effect size indicates a greater increase in UPCR in the eGFR < 60 mL/min/1.73 m^2^ group [21,23,30,36].

The rate of subsequent UPCR increase was also compared between the two eGFR categories (eGFR > 60 vs. eGFR < 60) within the male subgroup. The standardized effect sizes ranged from −0.123 to 0.106. This pooled analysis yielded a weighted mean effect size of 0.024, which did not reach statistical significance (*p* = 0.230) (Figure 7).

### 3.7. eGFR Decline Compared with UPCR < 0.5 and UPCR > 0.5

**Figure 8:**
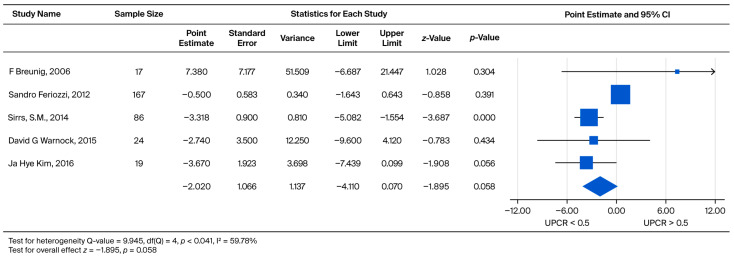
Subgroup analysis of annual eGFR slope in the overall cohort: comparison between patients with baseline UPCR < 0.5 and UPCR ≥ 0.5 g/g. A negative effect size indicates a faster eGFR decline in the UPCR ≥ 0.5 g/g group [21,26,28,30,37].

The prognostic relationship between UPCR and eGFR decline was assessed by grouping male and female patients into UPCR < 0.5 and UPCR > 0.5 cohorts. The pooled weighted mean effect size was calculated as −2.020. The observed difference was not statistically significant (*p* = 0.058), despite standardized measures ranging widely from −3.670 to 7.380 (Figure 8).

**Figure 9:**
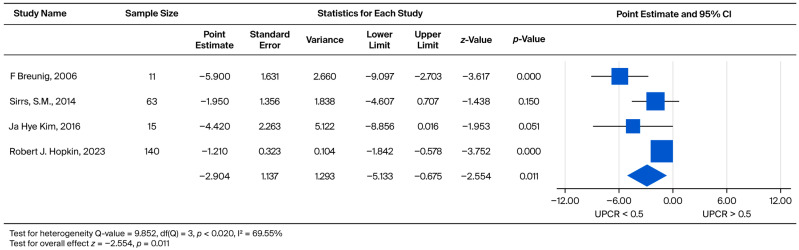
Subgroup analysis of annual eGFR slope in male patients: comparison between those with baseline UPCR < 0.5 and UPCR ≥ 0.5 g/g. A negative effect size indicates a faster eGFR decline in the UPCR ≥ 0.5 g/g group [21,30,34,37].

To determine the prognostic utility of proteinuria, male patients were separately analyzed based on baseline UPCR (UPCR < 0.5 vs. UPCR > 0.5). The pooled weighted mean effect size was found to be −2.904. Critically, this difference was statistically significant (*p* = 0.011), indicating a differential rate of decline between the two proteinuria levels (Figure 9).

### 3.8. UPCR Increase Compared with UPCR < 0.5 and UPCR > 0.5

**Figure 10:**
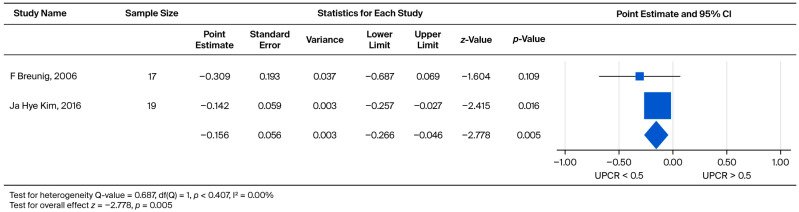
Subgroup analysis of annual UPCR change in the overall cohort: comparison between patients with baseline UPCR < 0.5 and UPCR ≥ 0.5 g/g. A negative effect size indicates a faster rate of UPCR progression in the UPCR ≥ 0.5 g/g group [21,30].

The rate of UPCR increase was significantly different (*p* = 0.005) between the UPCR > 0.5 and UPCR < 0.5 groups. The pooled weighted mean effect size was found to be −0.156, with standardized measures ranging from −0.309 to −0.142 (Figure 10).

**Figure 11:**
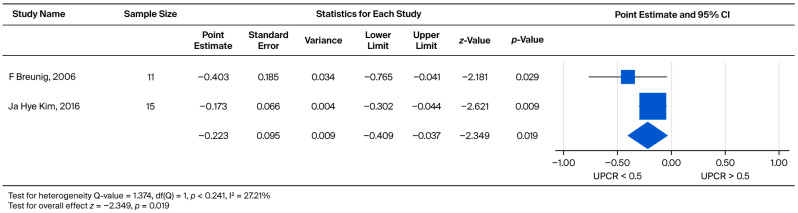
Subgroup analysis of annual UPCR change in male patients: comparison between those with baseline UPCR < 0.5 and UPCR ≥ 0.5 g/g. A negative effect size indicates a faster rate of UPCR progression in the UPCR ≥ 0.5 g/g group [21,30].

Furthermore, the impact of baseline proteinuria on the rate of UPCR increase was evaluated exclusively in the male cohort. The comparison yielded a pooled weighted mean effect size of −0.223. This effect was statistically significant (*p* = 0.019), demonstrating that higher baseline proteinuria is associated with a faster rate of subsequent proteinuria progression (Figure 11).

### 3.9. Clinical Events Compared with eGFR > 60 and eGFR < 60

**Figure 12:**
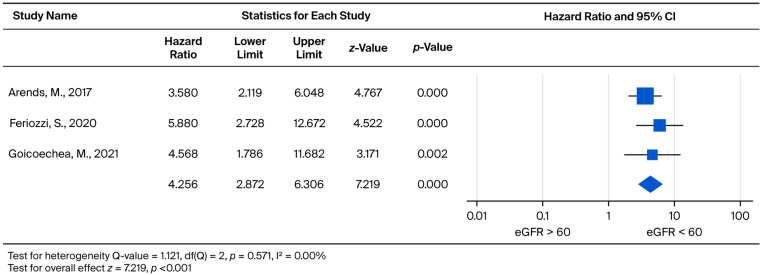
Forest plot comparing the risk of clinical events (hazard ratio) between patients with baseline eGFR ≥ 60 and eGFR < 60 mL/min/1.73 m^2^. A hazard ratio (HR) greater than 1 indicates a higher risk of clinical events in the eGFR < 60 mL/min/1.73 m^2^ group [31,32,33].

Analysis of clinical event risk by baseline eGFR stratification (eGFR > 60 vs. eGFR < 60) demonstrated a statistically significant increase in risk (*p* < 0.001) for the eGFR < 60 group. The treatment led to a 4.256-fold increased risk for clinical events (95% CI: 2.872 to 6.306) (Figure 12).

### 3.10. Assessment of Potential Publication Bias

To assess for small-study effects, we constructed Doi plots and calculated the Luis Furuya–Kanamori (LFK) index for all pooled meta-analyses comprising at least five studies (k ≥ 5). Overall, of the five analyses meeting this criterion, four demonstrated significant asymmetry (LFK Index absolute value ≥ 1), and one showed good symmetry.

Specifically, analyses for the Overall Annual eGFR Decline (Figure 2, k = 10) yielded an LFK Index of −5.43, indicating significant asymmetry. Similarly, significant asymmetry was observed in the comparison of eGFR > 60 vs. eGFR < 60 for the overall cohort (Figure 4, k = 9; LFK Index −4.29) and the male cohort (Figure 5, k = 8; LFK Index −4.64). The analysis for Male Annual eGFR Decline (Figure 3, k = 6) showed slight asymmetry (LFK Index −1.05). In contrast, the comparison of UPCR < 0.5 vs. UPCR ≥ 0.5 (Overall, Figure 8, k = 5) demonstrated good symmetry, with an LFK Index of 0.28. All Doi plots are presented in Supplementary Figure S2.

## 4. Discussion

In this systematic review and meta-analysis, we evaluated long-term renal outcomes in Fabry disease; both ERT-treated and untreated cohorts were included. Three main findings emerged. First, Fabry patients consistently experienced a decline in eGFR over time, regardless of treatment status. Second, while direct comparisons between the ERT and non-ERT groups rarely reached statistical significance, the trend favored earlier ERT initiation in patients with preserved renal function and lower proteinuria. Third, patients with baseline eGFR < 60 mL/min/1.73 m^2^ had a markedly higher risk of clinical events. These findings are consistent with clinical experience, in which advanced renal involvement often coincides with multi-organ complications. It is important to note that the forest plot summarizes within-cohort eGFR slopes rather than pairwise comparisons; therefore, the absence of a significant treatment effect in the meta-regression reflects study-level heterogeneity rather than a direct lack of therapeutic benefit.

These findings support the notion that ERT alone may not completely halt disease progression. Nevertheless, its benefits appear greater when treatment is initiated before substantial renal damage occurs. Moreover, our results emphasize the value of careful baseline risk stratification in daily clinical practice.

This systematic review and meta-analysis aimed to evaluate renal functional outcomes in patients with Fabry disease receiving long-term ERT. ERT has been used to treat Fabry disease for over a decade [15,16]. Tsuboi [38] and Tsurumi [39] suggested that, according to follow-up data, long-term use of agalsidase alpha and beta leads to few adverse effects. In addition, a report by Arends et al. [40] concluded that there is little difference between agalsidase alpha and beta in terms of effectiveness and safety [39]. Further, Cybulla et al. reported that patients who had undergone kidney transplantation could also safely receive ERT [41]. Several prior studies have indicated comparable efficacy and safety between agalsidase alpha and beta, although the available data remain limited. In our analysis, patients who received ERT exhibited a slower decline in eGFR compared with untreated individuals. However, the difference did not reach statistical significance. Given that the number of native patients was small, and due to ethical concerns, native patients may have transitioned to ERT treatment shortly after its approval. This could potentially cause bias, as these patients may have only remained untreated for a few months.

While our pooled analysis and the study by Arends et al. [31] suggest comparable overall eGFR declines between different ERT regimens, this remains a subject of debate. It is important to acknowledge the extensive work by Tøndel et al. [42] and Skrunes et al. [43], which demonstrated a dose-dependent effect for ERT. The findings of these studies indicate that higher doses of agalsidase may be more effective in clearing globotriaosylceramide (GL-3) inclusions from podocytes and may provide superior long-term renal protection, particularly in patients with more severe phenotypes or high antibody titers. Due to the limited number of studies suitable for quantitative synthesis, we were unable to perform a subgroup analysis stratified by enzyme dose, which represents a limitation of our study.

Although not statistically significant, our analysis showed a trend toward a faster eGFR decline rate in the untreated patient group compared to the treated group. The results were similar to those of Branton et al., who reported a mean rate of change in eGFR of −12.2 mL/min per year among untreated men with Fabry disease [8], and Prabakaran et al., who reported that the eGFR of untreated women was significantly decreased compared to the ERT-treated group [44]. In addition, Schiffmann et al. found that the rate of renal function declines faster and the frequency of clinical events increases in untreated patients [45]. A study by Nowak et al. found that the average eGFR slope in patients receiving ERT was noticeably lower than that of the placebo; however, the authors also concluded that ERT did not significantly influence the rate of eGFR decline [46]. Further, Madsen et al. reported that, in Fabry disease patients receiving ERT, only patients older than 50 years had a faster renal function loss than renal healthy subjects [47]. We believe that the reason for this is that the articles did not classify patients according to different baseline eGFR or UPCR levels, which could lead to different outcomes. Therefore, in our subsequent discussion, we will focus on patients with different levels of baseline renal function. We used an eGFR of 60 as the cutoff point, which is not only a commonly used threshold for identifying patients with stage 3 or higher CKD but also the dividing line in most of the articles we collected. Explicitly, Parini et al. have suggested that the earlier the use of ERT, the better the prognosis of patients [48].

Regarding the baseline renal function of patients, Wanner et al. noted that those with lower eGFR at baseline had a greater decline in eGFR during treatment [49]. In contrast, according to our findings, it appears that baseline eGFR was not associated with eGFR decline at follow-up after ERT. This held true for both males and females. The inconsistency may be explained by the different follow-up durations between the present review and prior reports. Whether patients on dialysis or those who had received a kidney transplant were excluded may have also influenced the outcomes. However, if we focus on UPCR, Lin et al. reported that proteinuria gradually decreased in some Fabry disease patients treated with ERT. However, despite this, patient eGFRs continue to deteriorate each year [35]. In addition, Germain et al. documented that the higher the patient’s baseline proteinuria, the faster the deterioration of the patient’s subsequent renal function [50]. Warnock et al. further believed that UPCR can be used as an indicator of renal outcomes [51]. Our meta-analysis demonstrated that Fabry disease patients with more severe baseline proteinuria exhibited a significantly faster rate of UPCR increase (*p* = 0.005 in the overall cohort and *p* = 0.019 in males; Figure 10 and Figure 11. This finding was further associated with a faster decline in eGFR, particularly in male patients (*p* = 0.011, Figure 9). Patients with lower urinary protein levels may continue to exhibit gradual kidney deterioration despite a reduction in proteinuria after treatment. Therefore, we used a two-by-two pairing of eGFR and UPCR to examine their combined effect. Based on these findings, initiating ERT in patients with limited kidney involvement (UPCR < 0.5) may yield the greatest benefit. Once glomerular injury occurs (UPCR > 0.5), the slope of eGFR decline becomes similar regardless of baseline function.

Ortiz et al. concluded that ERT could reduce the risk of clinical events [52]. Weidemann et al. reported that the worse the patient’s baseline renal function, the higher the chance of subsequent clinical events [53]. Wagner et al. also indicated that CKD, after the initiation of ERT but even early in disease progression, was a strong determinant of reduced quality of life in patients with Fabry disease [54]. However, to date, few studies have assessed the risk of clinical events based on different CKD stages. Our meta-analysis found that a baseline eGFR < 60 is associated with a greater risk of clinical events. Importantly, the study by Kim et al. suggested that ERT cannot recover organ function once damage has occurred [29]. Rombach et al. [24] and Germain et al. [55] also reported that renal function will continue to deteriorate (but at a slower rate) in response to ERT. Taken together, early initiation of ERT may not fully prevent eGFR decline but can reduce the frequency of multi-organ involvement and delay organ failure compared with late initiation. This observation may also explain the better therapeutic response in patients with lower baseline proteinuria.

Recent publications also help place our findings in the context of current Fabry disease research. Wanner et al. [56] reviewed more than 20 years of data from the Fabry Registry, showing that kidney function continues to decline over time, especially when treatment begins after kidney damage has occurred. These real-world observations are consistent with our results, highlighting the significant impact of baseline kidney status on long-term outcomes.

A recent systematic literature review by Jovanovic et al. [57] summarized renal, cardiac, and neurological outcomes across a wide range of studies. Because the included studies differed greatly in patient characteristics, follow-up duration, and outcome reporting, renal data could not be pooled into a single estimate. Our meta-analysis helps address this gap by providing a focused quantitative analysis of eGFR decline and by including subgroup analyses based on baseline eGFR and proteinuria. Together, these findings underscore the importance of early treatment initiation and careful evaluation of baseline renal status in Fabry disease.

To our knowledge, this is the first meta-analysis to evaluate and compare long-term renal function decline in patients with Fabry disease receiving enzyme replacement therapy (ERT) stratified by baseline eGFR and UPCR. By synthesizing evidence across diverse observational cohorts, this study provides a broader view of treatment response patterns that may not be apparent in individual studies. The inclusion of UPCR-based stratification further offers clinically meaningful insight into predictors of renal progression under ERT.

To evaluate the methodological robustness of our pooled estimates, we assessed for small-study effects by constructing Doi plots and calculating the LFK index for analyses where k ≥ 5. Our assessment revealed significant negative asymmetry in the analyses concerning the overall eGFR decline (LFK Index = −5.43) and eGFR baseline stratification (LFK Index ranging from −4.29 to −4.64). This negative asymmetry may suggest the presence of small-study effects or publication bias, where smaller studies tend to report larger negative effects (i.e., faster eGFR decline). In contrast, the analysis comparing UPCR baseline groups (Figure 8) showed good symmetry (LFK Index = 0.28). While we acknowledge the potential influence of publication bias, we confirmed the stability of our pooled results using a leave-one-out sensitivity analysis. These analyses consistently indicated that excluding any single study did not materially alter the overall effect sizes or our primary findings. Therefore, despite the observed asymmetry in eGFR-related outcomes, our key conclusion regarding the predictive value of baseline proteinuria and the importance of early intervention remains methodologically stable.

Several limitations of our meta-analysis should be noted. First, because Fabry disease is rare, the number of eligible studies and patients was limited; moreover, baseline renal function was inconsistently defined across studies. Furthermore, we acknowledge that classic and non-classic (later-onset) Fabry phenotypes exhibit distinct rates of disease progression. However, due to inconsistent reporting across the included studies, we were unable to perform a separate quantitative meta-analysis stratified by genetic phenotype. Consequently, our pooled results represent an aggregate of these phenotypes. To mitigate this, we performed subgroup analyses on the male population and patients with greater baseline severity (proteinuria and renal dysfunction), which serve as important clinical proxies for disease aggressiveness. Additionally, most cohorts excluded patients with ESRD or prior transplantation, potentially underestimating renal events. Our pooled analysis could also not differentiate between enzyme types or doses due to the heterogeneous nature of the reported data, which may mask potential differences in efficacy. Effect sizes were primarily expressed as standardized mean differences to accommodate heterogenous reporting, limiting direct clinical interpretability. The QUIPS tool, although suitable for prognostic studies, may not fully capture bias domains relevant to interventional designs. Finally, the most recent literature search was performed in October 2023, meaning that studies published thereafter were not included; however, this was consistent with the predefined methodological framework of this review.

## 5. Conclusions

This systematic review and meta-analysis demonstrates that patients with Fabry disease experience a progressive decline in renal function over time. Although direct comparisons between the ERT and non-ERT groups did not consistently reach statistical significance, our quantitative analysis highlighted baseline proteinuria as a major determinant of renal trajectory. Patients with baseline UPCR > 0.5 g/g exhibited a significantly faster decline in eGFR, particularly in the male cohort. Furthermore, patients with baseline eGFR < 60 mL/min/1.73 m^2^ carried a significantly greater risk of clinical events, underscoring the prognostic value of renal function at treatment initiation. Collectively, these findings highlight the need for timely diagnosis and early therapeutic intervention—specifically before the onset of significant proteinuria or renal impairment—to optimize long-term renal and clinical outcomes in Fabry disease.

## Figures and Tables

**Table 1:** Characteristics of included studies.

	Included Patient Number			Treatment	Follow-Up Duration			
	Male	Female	Mix			eGFR Slope	Proteinuria (UPCR)	Clinical Events
Mary H. Branton, 2002 [8]	105			naïve	data from 1970 to 2000	✓		
F Breunig, 2006 [21]			25	agalsidase beta	median of 23 months	✓	✓	
Raphael Schiffmann, 2006 [18]	24			agalsidase alpha	2 years	✓		
Anouk C. Vedder, 2007 [22]	12	16		agalsidase alpha or beta	24 months	✓		
Hindia Tahir, 2007 [23]			10	agalsidase beta	30 months	✓	✓	
Michael West, 2009 [24]	162			naïve, agalsidase alpha	48 months	✓		
Raphael Schiffmann, 2009 [25]	279	168		naïve	48 months	✓		
Sandro Feriozzi, 2012 [26]			208	agalsidase alpha	7.4 years (range: 5.0–11.2 years)	✓	✓	
Saskia M Rombach, 2013 [27]	30	27		agalsidase alpha	5.2 years (range: 0.05–11.0 years)	✓		
David G Warnock, 2015 [28]			24	agalsidase beta	21 months	✓		
Lubor Goláň, 2015 [29]			16	agalsidase alpha	53 weeks	✓		
Ja Hye Kim, 2016 [30]	15	4		agalsidase beta	median of 8.1 years	✓	✓	
Arends M, 2017 [31]	293			agalsidase alpha, beta	6.8 years (range: 0.8–15.4)	✓		✓
Feriozzi S, 2020 [32]	269	291		agalsidase alpha	180 months	✓		✓
Goicoechea M, 2021 [33]	42	27		agalsidase alpha, beta	60 months	✓		✓
Robert J. Hopkin, 2023 [34]	140			agalsidase beta	median of 6.3 years	✓		

A checkmark means the study includes this result.

**Table 2:** Subgroup analysis comparing eGFR decline rates between ERT-treated and non-ERT patients.

Subgroup	Point Estimate	SE	95%CI		Z-Value	*p*-Value
			Lower Limit	Upper Limit		
Intercept	−2.974	1.152	−5.231	−0.717	−2.580	0.010
ERT vs. no ERT	1.555	1.241	−0.878	3.987	1.250	0.210

**Table 3:** Subgroup analysis comparing annual eGFR slope between ERT-treated and untreated male patients.

Subgroup	Point Estimate	SE	95%CI		Z-Value	*p*-Value
			Lower Limit	Upper Limit		
Intercept	−3.387	0.432	−4.235	−2.540	−7.87	<0.001
ERT vs. no ERT	0.508	0.508	−0.487	1.503	1.00	0.317

## Data Availability

All data supporting the findings of this study are contained within the article and its Supplementary Materials. Additional details are available from the corresponding author upon reasonable request.

## Supplementary Materials

The following supporting information can be downloaded at https://www.mdpi.com/article/10.3390/biomedicines13122989/s1, Figure S1: the risk of bias in prognostic studies using QUIPS [8,18,21,22,23,24,25,26,27,28,29,30,31,32,33,34 ]; Table S1: PRISMA 2020 checklist for systematic reviews. Figure S2-1: Doi Plot for Overall Annual eGFR Decline (Corresponding to Main Manuscript Figure 2) LFK Index: −5.43; Figure S2-2: Doi Plot for Male Annual eGFR Decline (Corresponding to Main Manuscript Figure 3). LFK Index: −1.05; Figure S2-3: Doi Plot for eGFR > 60 vs. eGFR < 60 (Overall) (Corresponding to Main Manuscript Figure 4). LFK Index: −4.29; Figure S2-4: Doi Plot for eGFR > 60 vs. eGFR < 60 (Male) (Corresponding to Main Manuscript Figure 5). LFK Index: −4.64; Figure S2-5: Doi Plot for UPCR < 0.5 vs. UPCR ≥ 0.5 (Overall) (Corresponding to Main Manuscript Figure 8). LFK Index: 0.28 [37].

## Abbreviations

The following abbreviations are used in this manuscript:

ERT	Enzyme replacement therapy
eGFR	Estimated glomerular filtration rate
GFR	Glomerular filtration rate
Gb3	Globotriaosylceramide
CKD	Chronic kidney disease
UPCR	Urinary protein/creatinine ratio
EOW	Every other week
RCTs	Randomized controlled trials
PRISMA	Preferred Reporting Items for Systematic Reviews and Meta-Analyses
TIA	Transient ischemic attack
QUIPS	Quality in Prognosis Studies
SMD	Standardized mean difference
CI	Confidence interval
ESRD	End-stage renal disease
HR	Hazard ratio
α-Gal A	Alpha-galactosidase A
I2	Inconsistency index

## References

[1] Thompson S.E., Roy A., Geberhiwot T., Gehmlich K., Steeds R.P. (2025). Fabry Disease: Insights into Pathophysiology and Novel Therapeutic Strategies. Biomedicines.

[2] Huang Y., Yuan H., Huang Z. (2025). Cost-effectiveness analysis of enzyme replacement therapy for the treatment of Chinese patients with fabry disease: A Markov model. Front. Pharmacol..

[3] Germain D.P. (2010). Fabry disease. Orphanet J. Rare Dis..

[4] Bernardes T.P., Foresto R.D., Kirsztajn G.M. (2020). Fabry disease: Genetics, pathology, and treatment. Rev. Da Assoc. Médica Bras..

[5] Sugawara K., Ohno K., Saito S., Sakuraba H. (2008). Structural characterization of mutant alpha-galactosidases causing Fabry disease. J. Hum. Genet..

[6] Lin C.J., Chien Y.H., Lai T.S., Shih H.M., Chen Y.C., Pan C.F., Chen H.H., Hwu W.L., Wu C.J. (2018). Results of Fabry Disease Screening in Male Pre-End Stage Renal Disease Patients with Unknown Etiology Found Through the Platform of a Chronic Kidney Disease Education Program in a Northern Taiwan Medical Center. Kidney Blood Press. Res..

[7] Michaud M., Mauhin W., Belmatoug N., Garnotel R., Bedreddine N., Catros F., Ancellin S., Lidove O., Gaches F. (2020). When and How to Diagnose Fabry Disease in Clinical Pratice. Am. J. Med. Sci..

[8] Branton M.H., Schiffmann R., Sabnis S.G., Murray G.J., Quirk J.M., Altarescu G., Goldfarb L., Brady R.O., Balow J.E., Austin Iii H.A. (2002). Natural history of Fabry renal disease: Influence of alpha-galactosidase A activity and genetic mutations on clinical course. Medicine.

[9] Svarstad E., Marti H.P. (2020). The Changing Landscape of Fabry Disease. Clin. J. Am. Soc. Nephrol..

[10] Pisani A., Visciano B., Imbriaco M., Di Nuzzi A., Mancini A., Marchetiello C., Riccio E. (2014). The kidney in Fabry’s disease. Clin. Genet..

[11] Tapia D., Kimonis V. (2021). Stroke and Chronic Kidney Disease in Fabry Disease. J. Stroke Cerebrovasc. Dis..

[12] Chimenz R., Chirico V., Cuppari C., Ceravolo G., Concolino D., Monardo P., Lacquaniti A. (2022). Fabry disease and kidney involvement: Starting from childhood to understand the future. Pediatr. Nephrol..

[13] Del Pino M., Andres A., Bernabeu A.A., de Juan-Rivera J., Fernandez E., de Dios Garcia Diaz J., Hernandez D., Luno J., Fernandez I.M., Paniagua J. (2018). Fabry Nephropathy: An Evidence-Based Narrative Review. Kidney Blood Press. Res..

[14] Azevedo O., Gago M.F., Miltenberger-Miltenyi G., Sousa N., Cunha D. (2020). Fabry Disease Therapy: State-of-the-Art and Current Challenges. Int. J. Mol. Sci..

[15] van der Veen S.J., Hollak C.E.M., van Kuilenburg A.B.P., Langeveld M. (2020). Developments in the treatment of Fabry disease. J. Inherit. Metab. Dis..

[16] Lenders M., Brand E. (2021). Fabry Disease: The Current Treatment Landscape. Drugs.

[17] Banikazemi M., Bultas J., Waldek S., Wilcox W.R., Whitley C.B., McDonald M., Finkel R., Packman S., Bichet D.G., Warnock D.G. (2007). Agalsidase-beta therapy for advanced Fabry disease: A randomized trial. Ann. Intern. Med..

[18] Schiffmann R., Ries M., Timmons M., Flaherty J.T., Brady R.O. (2006). Long-term therapy with agalsidase alfa for Fabry disease: Safety and effects on renal function in a home infusion setting. Nephrol. Dial. Transplant..

[19] Schwarzer G., Rucker G., Semaca C. (2024). LFK index does not reliably detect small-study effects in meta-analysis: A simulation study. Res. Synth. Methods.

[20] Moher D., Liberati A., Tetzlaff J., Altman D.G. (2009). Preferred reporting items for systematic reviews and meta-analyses: The PRISMA statement. BMJ.

[21] Breunig F., Weidemann F., Strotmann J., Knoll A., Wanner C. (2006). Clinical benefit of enzyme replacement therapy in Fabry disease. Kidney Int..

[22] Vedder A.C., Linthorst G.E., Houge G., Groener J.E., Ormel E.E., Bouma B.J., Aerts J.M., Hirth A., Hollak C.E. (2007). Treatment of Fabry disease: Outcome of a comparative trial with agalsidase alfa or beta at a dose of 0.2 mg/kg. PLoS ONE.

[23] Tahir H., Jackson L.L., Warnock D.G. (2007). Antiproteinuric therapy and fabry nephropathy: Sustained reduction of proteinuria in patients receiving enzyme replacement therapy with agalsidase-beta. J. Am. Soc. Nephrol..

[24] West M., Nicholls K., Mehta A., Clarke J.T., Steiner R., Beck M., Barshop B.A., Rhead W., Mensah R., Ries M. (2009). Agalsidase alfa and kidney dysfunction in Fabry disease. J. Am. Soc. Nephrol..

[25] Schiffmann R., Warnock D.G., Banikazemi M., Bultas J., Linthorst G.E., Packman S., Sorensen S.A., Wilcox W.R., Desnick R.J. (2009). Fabry disease: Progression of nephropathy, and prevalence of cardiac and cerebrovascular events before enzyme replacement therapy. Nephrol. Dial. Transplant..

[26] Feriozzi S., Torras J., Cybulla M., Nicholls K., Sunder-Plassmann G., West M., on behalf of the FOS Investigators (2012). The effectiveness of long-term agalsidase alfa therapy in the treatment of Fabry nephropathy. Clin. J. Am. Soc. Nephrol..

[27] Rombach S.M., Hollak C.E., Linthorst G.E., Dijkgraaf M.G. (2013). Cost-effectiveness of enzyme replacement therapy for Fabry disease. Orphanet J. Rare Dis..

[28] Warnock D.G., Thomas C.P., Vujkovac B., Campbell R.C., Charrow J., Laney D.A., Jackson L.L., Wilcox W.R., Wanner C. (2015). Antiproteinuric therapy and Fabry nephropathy: Factors associated with preserved kidney function during agalsidase-beta therapy. J. Med. Genet..

[29] Golan L., Goker-Alpan O., Holida M., Kantola I., Klopotowski M., Kuusisto J., Linhart A., Musial J., Nicholls K., Gonzalez-Rodriguez D. (2015). Evaluation of the efficacy and safety of three dosing regimens of agalsidase alfa enzyme replacement therapy in adults with Fabry disease. Drug Des. Dev. Ther..

[30] Kim J.H., Lee B.H., Hyang Cho J., Kang E., Choi J.H., Kim G.H., Yoo H.W. (2016). Long-term enzyme replacement therapy for Fabry disease: Efficacy and unmet needs in cardiac and renal outcomes. J. Hum. Genet..

[31] Arends M., Wanner C., Hughes D., Mehta A., Oder D., Watkinson O.T., Elliott P.M., Linthorst G.E., Wijburg F.A., Biegstraaten M. (2017). Characterization of Classical and Nonclassical Fabry Disease: A Multicenter Study. J. Am. Soc. Nephrol..

[32] Feriozzi S., Linhart A., Ramaswami U., Kalampoki V., Gurevich A., Hughes D., on behalf of the Fabry Outcome Survey Study Group (2020). Effects of Baseline Left Ventricular Hypertrophy and Decreased Renal Function on Cardiovascular and Renal Outcomes in Patients with Fabry Disease Treated with Agalsidase Alfa: A Fabry Outcome Survey Study. Clin. Ther..

[33] Goicoechea M., Gomez-Preciado F., Benito S., Torras J., Torra R., Huerta A., Restrepo A., Ugalde J., Astudillo D.E., Agraz I. (2021). Predictors of outcome in a Spanish cohort of patients with Fabry disease on enzyme replacement therapy. Nefrol. (Engl. Ed.).

[34] Hopkin R.J., Cabrera G.H., Jefferies J.L., Yang M., Ponce E., Brand E., Feldt-Rasmussen U., Germain D.P., Guffon N., Jovanovic A. (2023). Clinical outcomes among young patients with Fabry disease who initiated agalsidase beta treatment before 30 years of age: An analysis from the Fabry Registry. Mol. Genet. Metab..

[35] Lin H.Y., Liu H.C., Huang Y.H., Liao H.C., Hsu T.R., Shen C.I., Li S.T., Li C.F., Lee L.H., Lee P.C. (2013). Effects of enzyme replacement therapy for cardiac-type Fabry patients with a Chinese hotspot late-onset Fabry mutation (IVS4+919G>A). BMJ Open.

[36] Sirrs S.M., Bichet D.G., Casey R., Clarke J.T.R., Lemoine K., Doucette S., West M.L., CFDI Investigators (2014). Outcomes of patients treated through the Canadian Fabry disease initiative. Mol. Genet. Metab..

[37] Page M.J., McKenzie J.E., Bossuyt P.M., Boutron I., Hoffmann T.C., Mulrow C.D., Shamseer L., Tetzlaff J.M., Akl E.A., Brennan S.E. (2021). The 14 PRISMA 2020 statement: An updated guideline for reporting systematic reviews. BMJ.

[38] Tsuboi K., Yamamoto H., Somura F., Goto H. (2015). Successful management of enzyme replacement therapy in related fabry disease patients with severe adverse events by switching from agalsidase Beta (fabrazyme^®^) to agalsidase alfa (replagal ^®^). JIMD Reports.

[39] Tsurumi M., Suzuki S., Hokugo J., Ueda K. (2021). Long-term safety and efficacy of agalsidase beta in Japanese patients with Fabry disease: Aggregate data from two post-authorization safety studies. Expert Opin. Drug Saf..

[40] Arends M., Biegstraaten M., Wanner C., Sirrs S., Mehta A., Elliott P.M., Oder D., Watkinson O.T., Bichet D.G., Khan A. (2018). Agalsidase alfa versus agalsidase beta for the treatment of Fabry disease: An international cohort study. J. Med. Genet..

[41] Cybulla M., Walter K.N., Schwarting A., Divito R., Feriozzi S., Sunder-Plassmann G., on behalf of the European FOS Investigators Group (2009). Kidney transplantation in patients with Fabry disease. Transpl. Int..

[42] Tondel C., Bostad L., Larsen K.K., Hirth A., Vikse B.E., Houge G., Svarstad E. (2013). Agalsidase benefits renal histology in young patients with Fabry disease. J. Am. Soc. Nephrol..

[43] Skrunes R., Tondel C., Leh S., Larsen K.K., Houge G., Davidsen E.S., Hollak C., van Kuilenburg A.B.P., Vaz F.M., Svarstad E. (2017). Long-Term Dose-Dependent Agalsidase Effects on Kidney Histology in Fabry Disease. Clin. J. Am. Soc. Nephrol..

[44] Prabakaran T., Birn H., Bibby B.M., Regeniter A., Sorensen S.S., Feldt-Rasmussen U., Nielsen R., Christensen E.I. (2014). Long-term enzyme replacement therapy is associated with reduced proteinuria and preserved proximal tubular function in women with Fabry disease. Nephrol. Dial. Transplant..

[45] Schiffmann R. (2015). Fabry disease. Handb. Clin. Neurol..

[46] Nowak A., Koch G., Huynh-Do U., Siegenthaler M., Marti H.P., Pfister M. (2017). Disease Progression Modeling to Evaluate the Effects of Enzyme Replacement Therapy on Kidney Function in Adult Patients with the Classic Phenotype of Fabry Disease. Kidney Blood Press. Res..

[47] Madsen C.V., Granqvist H., Petersen J.H., Rasmussen A.K., Lund A.M., Oturai P., Sorensen S.S., Feldt-Rasmussen U. (2019). Age-related renal function decline in Fabry disease patients on enzyme replacement therapy: A longitudinal cohort study. Nephrol. Dial. Transplant..

[48] Parini R., Pintos-Morell G., Hennermann J.B., Hsu T.R., Karabul N., Kalampoki V., Gurevich A., Ramaswami U., FOS Study Group (2020). Analysis of Renal and Cardiac Outcomes in Male Participants in the Fabry Outcome Survey Starting Agalsidase Alfa Enzyme Replacement Therapy Before and After 18 Years of Age. Drug Des. Dev. Ther..

[49] Wanner C., Feldt-Rasmussen U., Jovanovic A., Linhart A., Yang M., Ponce E., Brand E., Germain D.P., Hughes D.A., Jefferies J.L. (2020). Cardiomyopathy and kidney function in agalsidase beta-treated female Fabry patients: A pre-treatment vs. post-treatment analysis. ESC Heart Fail..

[50] Germain D.P., Waldek S., Banikazemi M., Bushinsky D.A., Charrow J., Desnick R.J., Lee P., Loew T., Vedder A.C., Abichandani R. (2007). Sustained, long-term renal stabilization after 54 months of agalsidase beta therapy in patients with Fabry disease. J. Am. Soc. Nephrol..

[51] Warnock D.G., Ortiz A., Mauer M., Linthorst G.E., Oliveira J.P., Serra A.L., Marodi L., Mignani R., Vujkovac B., Beitner-Johnson D. (2012). Renal outcomes of agalsidase beta treatment for Fabry disease: Role of proteinuria and timing of treatment initiation. Nephrol. Dial. Transplant..

[52] Ortiz A., Abiose A., Bichet D.G., Cabrera G., Charrow J., Germain D.P., Hopkin R.J., Jovanovic A., Linhart A., Maruti S.S. (2016). Time to treatment benefit for adult patients with Fabry disease receiving agalsidase beta: Data from the Fabry Registry. J. Med. Genet..

[53] Weidemann F., Niemann M., Stork S., Breunig F., Beer M., Sommer C., Herrmann S., Ertl G., Wanner C. (2013). Long-term outcome of enzyme-replacement therapy in advanced Fabry disease: Evidence for disease progression towards serious complications. J. Intern. Med..

[54] Wagner M., Krämer J., Blohm E., Vergho D., Weidemann F., Breunig F., Wanner C. (2014). Kidney function as an underestimated factor for reduced health related quality of life in patients with Fabry disease. BMC Nephrol..

[55] Germain D.P., Charrow J., Desnick R.J., Guffon N., Kempf J., Lachmann R.H., Lemay R., Linthorst G.E., Packman S., Scott C.R. (2015). Ten-year outcome of enzyme replacement therapy with agalsidase beta in patients with Fabry disease. J. Med. Genet..

[56] Wanner C., Ortiz A., Wilcox W.R., Hopkin R.J., Johnson J., Ponce E., Ebels J.T., Batista J.L., Maski M., Politei J.M. (2023). Global reach of over 20 years of experience in the patient-centered Fabry Registry: Advancement of Fabry disease expertise and dissemination of real-world evidence to the Fabry community. Mol. Genet. Metab..

[57] Jovanovic A., Miller-Hodges E., Castriota F., Evuarherhe O., Ayodele O., Hughes D., Pintos-Morell G., Giugliani R., Feriozzi S., Siffel C. (2025). Clinical Efficacy and Real-World Effectiveness of Fabry Disease Treatments: A Systematic Literature Review. J. Clin. Med..

