# Supplementary material for: Long-Term Enzyme Replacement Therapy and Renal Outcomes in Fabry Disease: A Systematic Review and Meta-Analysis
Source: Biomedicines. 2025 Dec 5;13(12):2989. doi: 10.3390/biomedicines13122989 (PMC12730587; doi:10.3390/biomedicines13122989)
Supplement: Supplementary file 1 [file biomedicines-13-02989-s001.zip › biomedicines-3944910-supplementary 2.pdf]

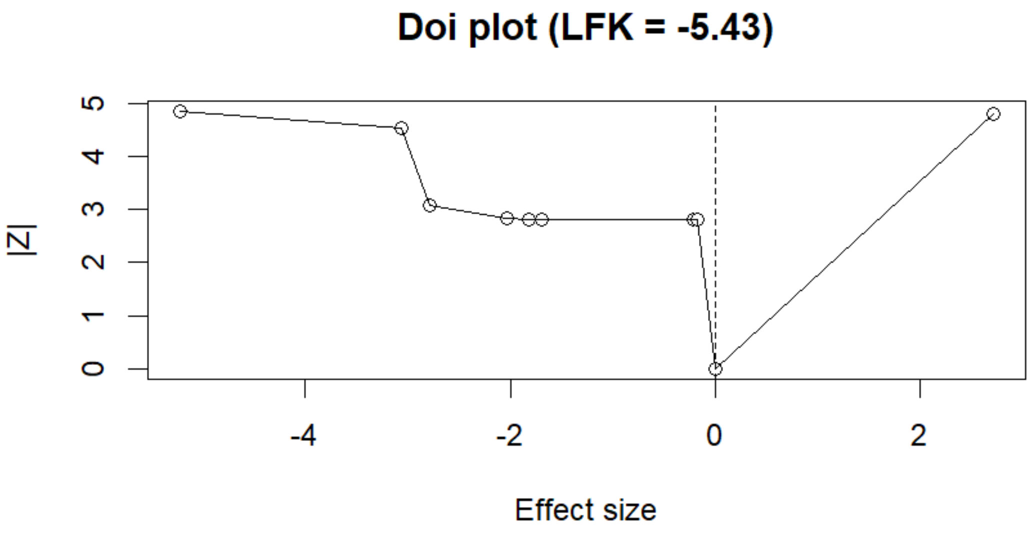

Figure S2-1. Doi Plot for Overall Annual eGFR Decline (Corresponding to Main Manuscript Figure 2) LFK Index: -5.43.

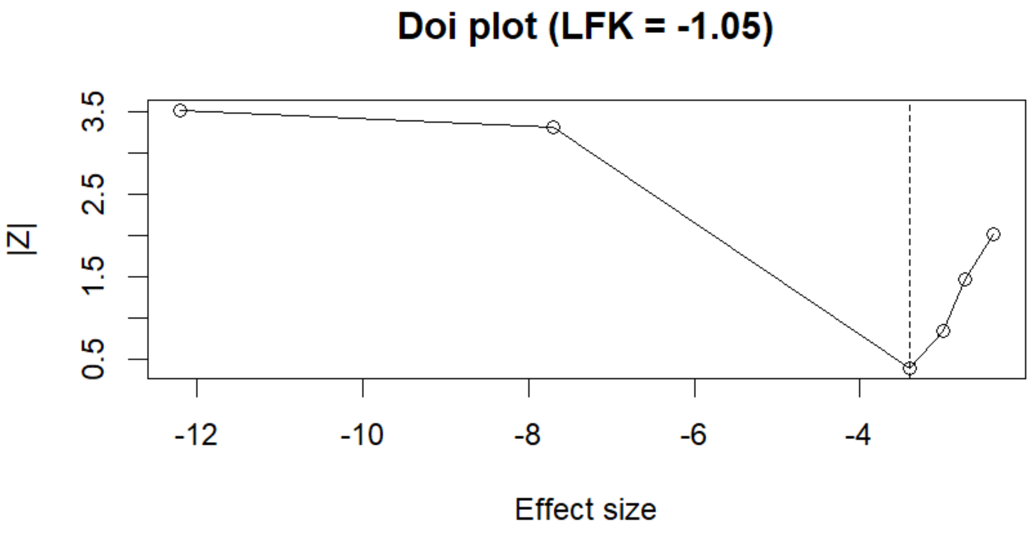

Figure S2-2. Doi Plot for Male Annual eGFR Decline (Corresponding to Main Manuscript Figure 3). LFK Index: -1.05.

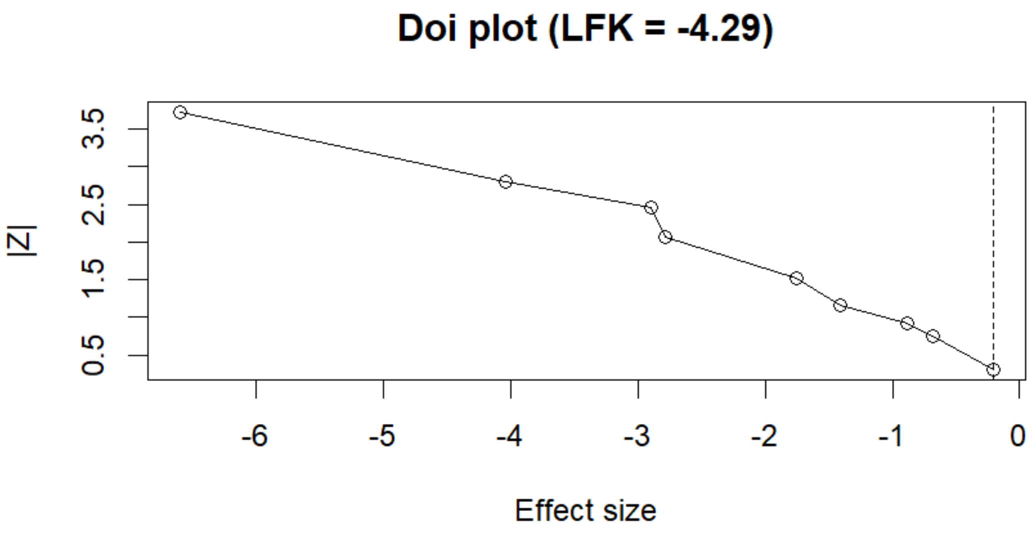

Figure S2-3. Doi Plot for eGFR > 60 vs. eGFR < 60 (Overall) (Corresponding to Main Manuscript Figure 4). LFK Index: -4.29.

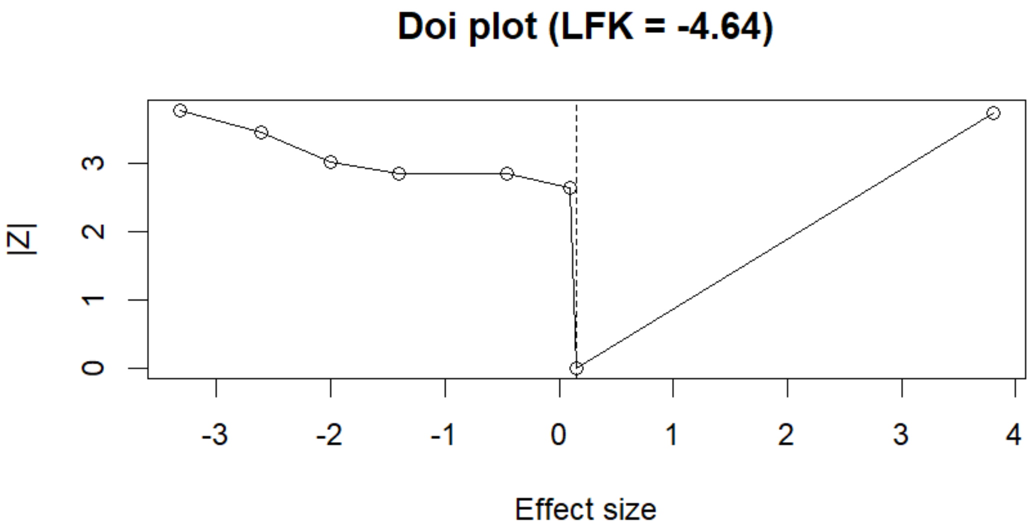

Figure S2-4. Doi Plot for eGFR > 60 vs. eGFR < 60 (Male) (Corresponding to Main Manuscript Figure 5). LFK Index: -4.64.

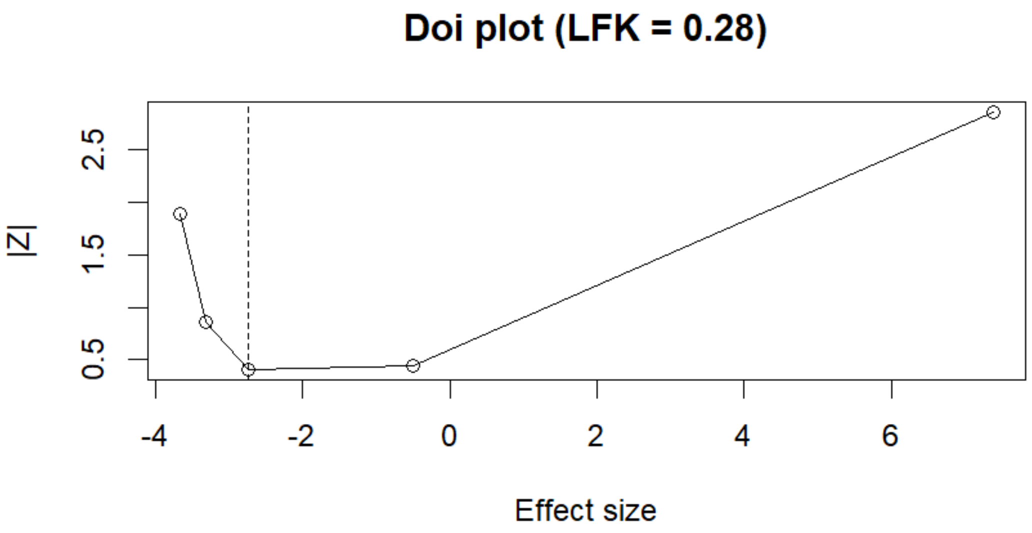

Figure S2-5. Doi Plot for UPCR < 0.5 vs. UPCR ≥ 0.5 (Overall) (Corresponding to Main Manuscript Figure 8). LFK Index: 0.28.
